# Supplementary material for: Comparative multiomics analysis of cell physiological state after culture in a basket bioreactor
Source: Sci Rep. 2022 Nov 23;12:20161. doi: 10.1038/s41598-022-24687-4 (PMC9686226; doi:10.1038/s41598-022-24687-4)
Supplement: Supplementary file 1 — Supplementary Information 1. [file 41598_2022_24687_MOESM1_ESM.zip › raw data/Transcriptomic raw data/7.AS/2.dasplot/TE1vsTC1/A5SS/4_EPN1_NW_023666045.1_924479_924540_+@NW_023666045.1_924479_924669_+@NW_023666045.1_925958_926306_+.pdf]

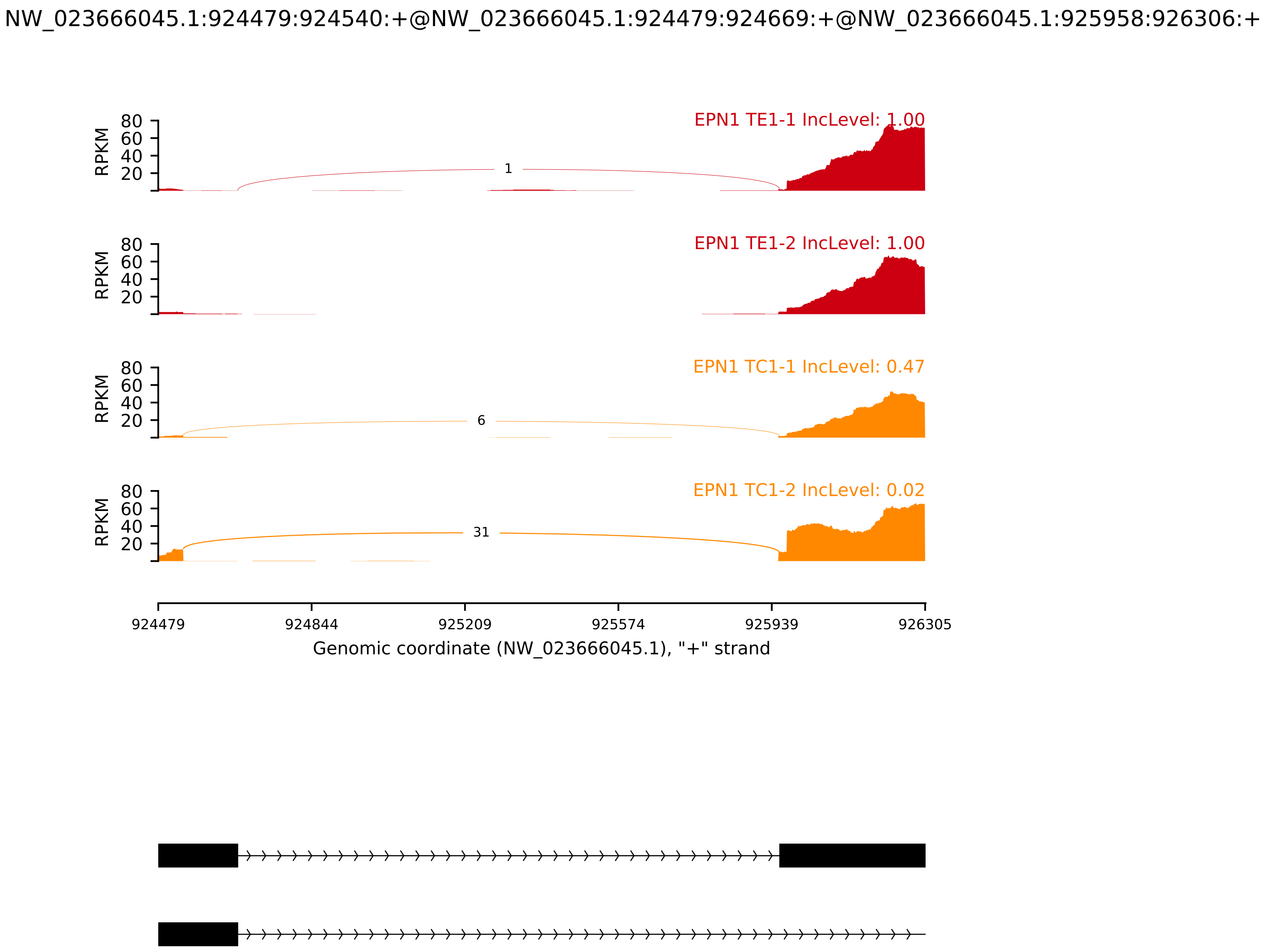
NW_023666045.1:924479:924540:+@NW_023666045.1:924479:924669:+@NW_023666045.1:925958:926306:+
EPN1 TE1-1 IncLevel: 1.00
EPN1 TE1-2 IncLevel: 1.00
EPN1 TC1-1 IncLevel: 0.47
EPN1 TC1-2 IncLevel: 0.02
RPKM
80
60
40
20
1
6
31
924479
924844
925209
925574
925939
926305
Genomic coordinate (NW_023666045.1), "+" strand
